# The cost impact of PCT-guided antibiotic stewardship versus usual care for hospitalised patients with suspected sepsis or lower respiratory tract infections in the US: A health economic model analysis

**DOI:** 10.1371/journal.pone.0214222

**Published:** 2019-04-23

**Authors:** Janne C. Mewes, Michael S. Pulia, Michael K. Mansour, Michael R. Broyles, H. Bryant Nguyen, Lotte M. Steuten

**Affiliations:** 1 Panaxea B.V., Amsterdam, the Netherlands; 2 BerbeeWalsh Department of Emergency Medicine, University of Wisconsin-Madison School of Medicine and Public Health, Madison, WI, United States of America; 3 Division of Infectious Diseases, Department of Medicine, Massachusetts General Hospital, Boston, MA, United States of America; 4 Harvard Medical School, Boston, MA, United States of America; 5 Department of Clinical Pharmacy and Laboratory Services, Five Rivers Medical Center, Pocahontas, AR, United States of America; 6 Division of Pulmonary, Critical Care, Hyperbaric and Sleep Medicine, Loma Linda University, Loma Linda, CA, United States of America; 7 Department of Emergency Medicine, Loma Linda University, Loma Linda, CA, United States of America; 8 Fred Hutchinson Cancer Research Center, Hutchinson Institute for Cancer Outcomes Research, Seattle, WA, United States of America; 9 University of Washington, School of Pharmacy, the CHOICE Institute, Seattle, WA, United States of America; Azienda Ospedaliero Universitaria Careggi, ITALY

## Abstract

**Background:**

Procalcitonin is a biomarker that supports clinical decision-making on when to initiate and discontinue antibiotic therapy. Several cost (-effectiveness) analyses have been conducted on Procalcitonin-guided antibiotic stewardship, but none mainly based on US originated data.

**Objective:**

To compare effectiveness and costs of a Procalcitonin-algorithm versus standard care to guide antibiotic prescription for patients hospitalized with a diagnosis of suspected sepsis or lower respiratory tract infection in the US.

**Methods:**

A previously published health economic decision model was used to compare the costs and effects of Procalcitonin-guided care. The analysis considered the societal and hospital perspective with a time horizon covering the length of hospital stay. The main outcomes were total costs per patient, including treatment costs and productivity losses, the number of patients with antibiotic resistance or *C*.*difficile* infections, and costs per antibiotic day avoided.

**Results:**

Procalcitonin -guided care for hospitalized patients with suspected sepsis and lower respiratory tract infection is associated with a reduction in antibiotic days, a shorter length of stay on the regular ward and the intensive care unit, shorter duration of mechanical ventilation, and fewer patients at risk for antibiotic resistant or *C*.*difficile* infection. Total costs in the Procalcitonin-group compared to standard care were reduced by 26.0% in sepsis and 17.7% in lower respiratory tract infection (total incremental costs of −$11,311 per patient and −$2,867 per patient respectively).

**Conclusions:**

Using a Procalcitonin-algorithm to guide antibiotic use in sepsis and hospitalised lower respiratory tract infection patients is expected to generate cost-savings to the hospital and lower rates of antibiotic resistance and *C*.*difficile* infections.

## Background

Antibiotics are essential for treating sepsis and lower respiratory tract infections (LRTI), including pneumonia and COPD exacerbations. Yet, inappropriate prescribing is pervasive and contributes to the development of antibiotic resistance (ABR) and *C*.*difficile* infections, and poses an increasing burden on healthcare resources [1, 2].

Antibiotic resistance is considered one of the most pressing health threats globally. In the US, every year 2 million people acquire a bacterial infection that is resistant to at least one antibiotic, causing 23,000 deaths [1]. The healthcare burden of *C*.*difficile* infections has also increased during the last decade, as it is becoming more frequent, while at the same time harder to treat [1].

Biomarkers to guide antibiotic treatment decisions have been proposed as an effective and efficient way to enhance the appropriate use of antibiotics. As a biomarker, procalcitonin (PCT) has good specificity to distinguish bacterial from non-bacterial inflammations and could therefore help prevent unnecessary antibiotic prescriptions and/or reduce the duration of antibiotic therapy [2]. PCT can be detected in the blood 3–4 hours after a bacterial stimulus and inflammatory response. PCT reaches its highest concentration after 14–25 hours and has a half-life of 22–35 hours after the bacterial stimulus has been appropriately treated and the inflammatory response begins to resolve. Using a PCT-assay and a PCT-algorithm, clinicians can make appropriate decisions regarding antibiotic usage [3]. Meta-analyses showed that PCT is safe and effective, resulting in reduced antibiotic initiation and shorter antibiotic duration [4]. These benefits however, come at the additional costs of PCT-testing. Cost-effectiveness analyses are therefore needed to evaluate to what extent the health benefits, and their associated cost savings, can offset the added costs of PCT-testing. Several cost-effectiveness analyses based on European data [5–7] have shown increased costs for obtaining PCT-levels, and net savings when considering the downstream cost impacts of PCT-guided ABS on resource utilization. The aim of this paper is to conduct an economic evaluation of PCT from a US societal perspective, using US data when available. Our analyses incorporate direct medical costs, costs of *C*.*difficile* infections and antibiotic resistance, as well as productivity losses.

## Methods

**Fig 1:**
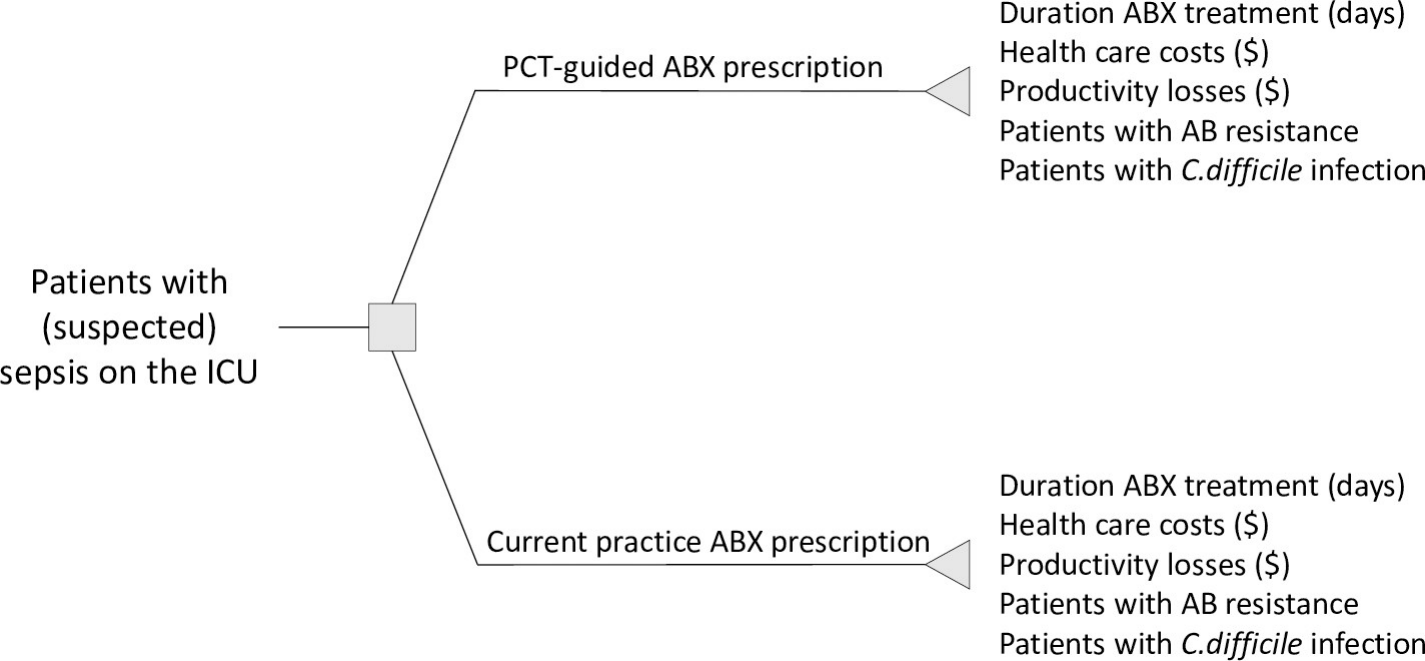
Decision-tree for patients with sepsis. ABX = Antibiotics.

**Fig 2:**
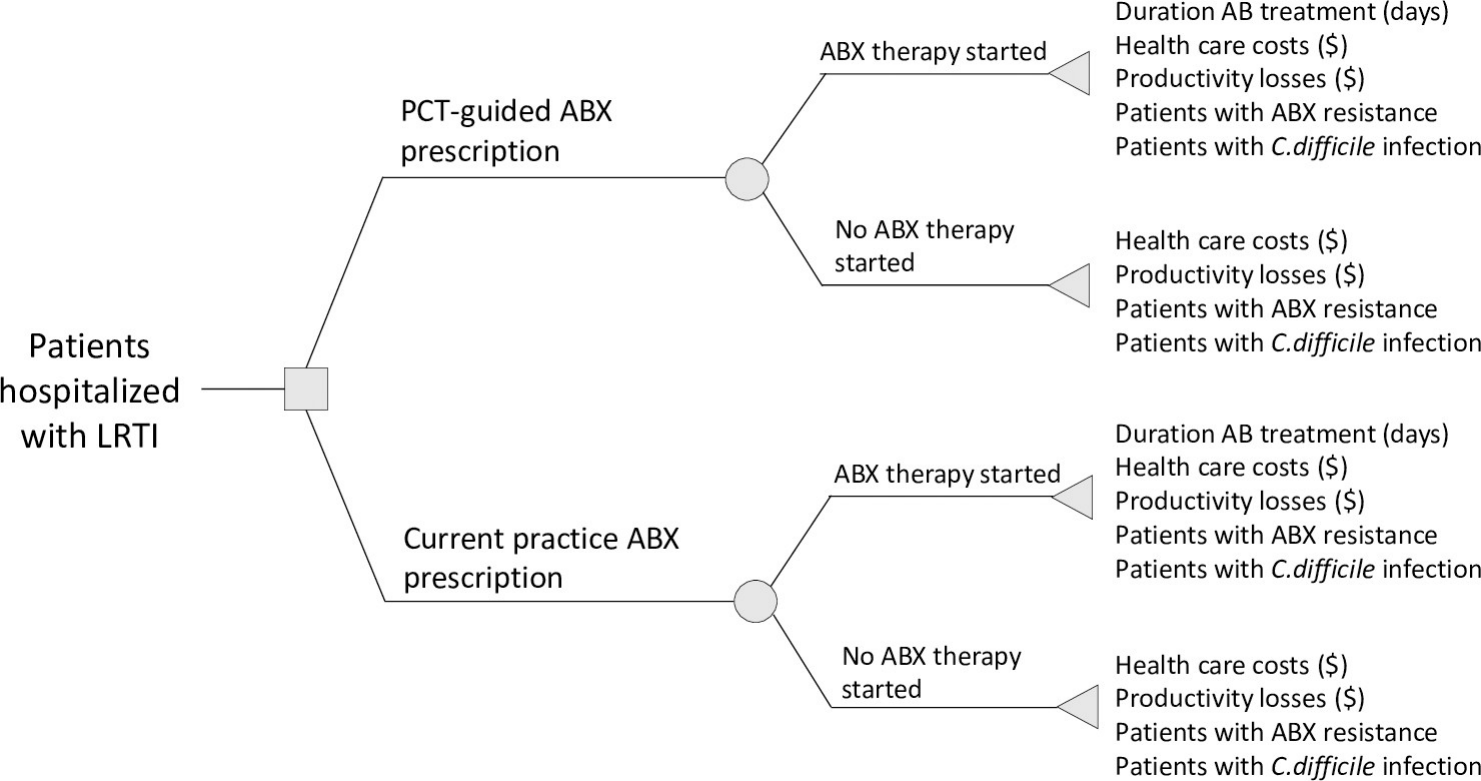
Decision-tree for patients with LRTI. ABX = Antibiotics.

This literature-based study is a comparative effectiveness and costs analysis of PCT-guided antibiotic stewardship versus standard care for patients with suspected sepsis on the intensive care unit (ICU) and patients hospitalized with LRTI, using a previously published decision tree model [8], as shown in Figs 1 and 2.

The difference between the analysis for sepsis and LRTI and the reason they are presented separately is that the model assumes that all sepsis patients are initially admitted to the ICU and all receive antibiotics initially, which is not the case for LRTI patients. The analysis was conducted from the societal perspective using a time horizon covering the length of the hospital stay.

In PCT-guided care, an algorithm was used to guide the decision of whether to start antibiotic treatment (LRTI) and when to stop administering antibiotics (LRTI and sepsis). Standard care included all usual care but no usage of a PCT-algorithm to guide the decision on when to start or stop antibiotics. The patient population consisted of patients with suspected sepsis in the ICU and patients hospitalised with LRTI. The latter included patients with COPD exacerbations, community-acquired pneumonia (CAP), ventilator-associated pneumonia (VAP), and acute bronchitis. In the model, the yearly number of patients hospitalised in the US with suspected sepsis or LRTI patients was included; 950,000 sepsis patients [9] and 1.9m LRTI patients [10–12].

The main outcome of the analysis were 1) the total difference in costs per patient and by patient population, 2) number of antibiotic days avoided, 3) number of *C*.*difficile* infections avoided, and 4) number of antibiotic resistance cases avoided. We also reported the incremental costs per antibiotic day avoided. All outcomes were analysed separately for (suspected) sepsis and LRTI patients.

### Data collection

A systematic literature review was conducted to identify studies examining the effectiveness of PCT-guided antibiotic therapy versus standard care. Pubmed was searched using the following search terms: (LRTI OR sepsis OR septic shock OR critically ill OR pneumonia OR bronchitis OR COPD OR bacterial infection) AND (PCT or procalcitonin) AND (antibiotic* OR antimicrobial OR "antibiotic stewardship" OR "antibiotic therapy"). Studies to be included had to be published from the year 2000 or later, be conducted in the hospital setting, include adult patients with (suspected) sepsis or LRTI, compare the effect of using a PCT-algorithm for guiding antibiotic therapy to standard care, and contain data on the effect of PCT-guided care on the number of antibiotic days, number of physician visits, hospital length of stay (regular ward and ICU), percentage of patients and number of days on mechanical ventilation, or rates of *C*.*difficile* infections and antibiotic resistant infections. Unsystematic and narrative reviews were excluded. US studies were prioritized for inclusion. In case no US data were available, non-US studies were considered, after validating the data’s applicability to the US healthcare setting by US clinician-researchers (MP, MK, MB, and BN). Data that could not be identified in any of the studies included in the literature review was taken from the previous model [8].

The selection of studies was conducted by JCM, MSP, and HBN. Titles and abstracts from our Pubmed search were screened, and full texts were obtained for those meeting our inclusion criteria. Selection was done by consensus. By manually reviewing reference lists of included studies additional citations were identified and included if appropriate.

If US or multi-national studies including US data were available, then US data were extracted from those studies using standardized forms; otherwise European data were extracted. For studies that did not report US-specific estimates, the authors were contacted and asked to share the US-specific data. When two or more studies provided data on one parameter, the weighted averages were used. For this purpose, results that were presented as medians and ranges were converted to means and standard deviations. Data on antibiotic use that were presented as days of therapy were converted into antibiotic days, based on the average number of antibiotics used per day requested from the authors. Cost were obtained from published studies and online reports, CMS Fee Schedules, and public databases on health care costs.

### Input data

The key input parameters for the effectiveness of PCT-guided antibiotic use, including length of stay on the regular ward and on the ICU (days), number of antibiotic days, and duration of mechanical ventilation (days), were derived exclusively from US studies [13–16]. The reduction in antibiotic resistant infections due to PCT-guided antibiotic management was determined by first conservatively estimating the prevalence of ABR in the U.S. population. This was 21.7% in sepsis and 22.2% in LRTI [17]. Based on previously published studies a correlation between the percentage of reduction in antibiotic days and ABR rate of 3.2% was established [18–20] (Steuten, Mewes et al–forthcoming). This rate was multiplied with the reduction in ABR based on the percentage of reduction in antibiotic days (the difference in antibiotic days between the PCT-guided group and usual care divided by the number of antibiotic days in the usual care group) [18–20].

The number of mechanical ventilation days was analysed by multiplying the mechanical ventilation days per 1,000 patient days by length of stay on the ICU [14]. For patients with LRTI, mechanical ventilation days were only applicable for the patients who were admitted to the ICU.

We used previously published European data to estimate the proportion of (suspected) sepsis patients with blood cultures performed and sets of blood cultures taken, number of other laboratory tests performed, and number of PCT measurements. These were confirmed by the co-authors to be similar with US clinical practice. For all effectiveness input parameters and resource use see Tables 1 and 2.

**Table 1:** Effectiveness input data.

Parameter	Standard care	PCT-guided strategy	Source	Country
**Sepsis**				
Number of days on the regular ward	5.80	5.10	Bishop et al., 2014 [14]	US
Number of days on the ICU	12.00	8.40	Bishop et al., 2014 [14]	US
Number of days on antibiotic therapy	13.37	7.54	Weighted average of the antibiotic days of Bishop et al, 2014 [14] and Broyles, 2017 [15]. Both adapted for the number of sepsis patients included in the analysis. Of Broyles, 2017 [15] the data specifically for sepsis patients was obtained.	US
Number of mechanical ventilation days	5.50	3.50	Bishop et al., 2014 [14]. Days on mechanical ventilation/1,000 patient days multiplied with the length of stay on the ICU.	US
Hospital *C*.*difficile* infections	3.1%	1.4%	Weighted average of Broyles, 2017 [15] and Bishop et al., 2014 [14]	US
Baseline antibiotic resistance infections rate in the general population	21.7%		The Center for Disease Dynamics, Economics, and Policy, 2017 [17]	US
Reduction in antibiotic resistance infections	3.2%		Chastre et al., 2003 [18], Singh et al., 2000 [19], Van der Maas [20]. This percentage is multiplied with the percentage of reduction in antibiotic days (the difference in antibiotic days divided by the antibiotic days in usual care).	France, US
Additional days on the general ward of a patient who develops antibiotic resistance infection	4.6		Mitchell et al., 2012 [21]	US
Additional days on the general ward of a patient with *C*.*difficile* infection	8.49		De Kraker et al., 2011 [21], Roberts et al., 2009 [23], and Lye et al., 2011 [24]	European countries, US, Singapore
**LRTI**				
Number of regular ward days	5.80	5.10	Bishop et al., 2014 [14]	US
Number of ICU days (applicable only for patients who are on the ICU)	12.00	8.40	Bishop et al., 2014 [14]	US
Proportion of patients admitted to ICU	10.5%	10.5%	Albrich et al., 2012 [5]	Multi-centre trial: France, Switzerland, US
Proportion of patients treated with antibiotics, %	87.7%	75.4%	Schuetz et al., 2009 [25]	Switzerland
Number of days on antibiotic therapy	11.90	6.99	Weighted average of the antibiotic days of Kook et al., 2012 and Broyles, 2017 [13, 15]. For Broyles, 2017 the data specifically for LRTI was obtained from the author.	US
Number of mechanical ventilation days of LRTI patients who are on the ICU	5.50	3.50	Bishop et al., 2014 [14]. Days on mechanical ventilation/1,000 patient days multiplied with the length of stay on the ICU.	US
Hospital *C*.*difficile* infections	3.1%	1.4%	Weighted average of Broyles, 2017 [15] and Bishop et al., 2014 [14]	US
Baseline antibiotic resistance infection rate	22.2%		The Center for Disease Dynamics, Economics, and Policy, 2017 [17]	US
Reduction in antibiotic resistance infections	3.2%		Chastre et al., 2003 [18] and Singh et al., 2000 [19], Van der Maas [20]. This percentage is multiplied with the percentage of reduction in antibiotic days (the difference in antibiotic days divided by the antibiotic days in usual care).	France, US
Additional days on the general ward of a patient who develops antibiotic resistance infection	8.1		Mitchell et al., 2012 [21]	US
Additional days on the general ward of a patient with *C*.*difficile* infection	8.49		De Kraker et al., 2011 [22], Roberts et al., 2009 [23], and Lye et al., 2011 [24]	European countries, US, Singapore

**Table 2:** Resource use.

Parameter	Standard care	PCT-guided strategy	Source	Country
Proportion of patients with blood culture taken	97.5%	61.4%	Müller et al., 2010 [26]	Switzerland
Proportion of patients with blood culture performed, diagnosed as having sepsis (applicable to sepsis patients only)	8.2%	8.2%	Shapiro et al., 2008 [27]	US
Sets of blood cultures taken per patient	2	2	Müller et al., 2010 [26]	Switzerland
Number of other laboratory tests ordered per patient	21.80	25.10	Kip et al., 2015 [8]	Switzerland
Number of PCT measurements performed per patient	0	5	Schuetz et al., 2009 [25]	Switzerland
NAAT taken of patients with *C*.*difficile* infection	1	1	McDonald et al., 2018 [28]	US
GDH taken of patients with *C*.*difficile* infection	1	1	McDonald et al., 2018 [28]	US
Common antigen immunoassay taken of patients with *C*.*difficile* infection	1	1	McDonald et al., 2018 [28]	US

Cost categories included in the decision tree model pertained to costs of hospital stay, treatment costs (including antibiotics and mechanical ventilation), laboratory analyses (including blood cultures, PCT tests, and other laboratory tests), antibiotic resistant infections, *C*.*difficile* infections, and productivity losses. The latter were calculated by multiplying the number of days in hospital by the productivity costs for an 8-hour workday. Costs of *C*.*difficile* infections and ABR consisted of costs for extended length of stay and additional blood and diagnostic tests during the extended stay.

All costs were based on US-data and converted to 2017 US dollars using the CPI inflation calculator. Discounting was not required as the time horizon of the model is shorter than one year. See Table 3 for the cost inputs.

**Table 3:** Cost input data for sepsis and LRTI, all based on the US.

Parameter	Value	Source	Country
Hospital stay general ward, per day	$1,270.58	Balk et al., 2017 [29]	US
Hospital stay ICU, per day	$1,893.15	Kaiser State Health Facts, 2015 [30]	US
Day on mechanical ventilation	$1,050.00	CMS Fee Schedule [31]	US
Costs per day antibiotic therapy	Sepsis: $57.13 LRTI: $56.16	Balk et al., 2017 [29] Calculation based on Kalil et al., 2016 [32] and Drugs.com [33]	US
Set of blood cultures performed	$19.14	CMS Fee Schedule [31]	US
Other lab tests	$50.00	Assumption	US
PCT test	$49.66	Clinical Laboratory Fee Schedule [34]	US
Hospital stay in isolation, per day	$50	Assumption, the $50 are added hospital stay costs	US
Productivity costs per hour	$21.20	Neumann et al., 2016 [35]	US
NAAT test	$48.14	Clinical Laboratory Fee Schedule [34]	US
GDH	$10.60	Clinical Laboratory Fee Schedule [34]	US
Common antigen immunoassay	$20.56	Clinical Laboratory Fee Schedule [34]	US

### Analysis

In the decision tree, antibiotic days avoided were evaluated for two treatment pathways, PCT-guided antibiotic use and standard care. Costs were assigned to each pathway in the model. For every cost item included (costs of the hospital stay, antibiotic treatment, mechanical ventilation, laboratory analyses including PCT test, costs of antibiotic resistance and *C*.*difficile* infections, and productivity losses) the costs were calculated by multiplying the volumes used by the unit costs. This was done for every cost item and finally added up into a total cost for PCT-guided care and a total cost for standard care. Incremental costs per patient were calculated by subtracting the costs for the standard care strategy from the expected costs of the PCT-guided strategy. Total costs on a population level were analysed by multiplying the annual number of hospitalised patients with (suspected) sepsis or LRTI with the expected total costs per patient for each strategy. The number of patients with *C*.*difficile* infection or antibiotic resistant infection were estimated by multiplying the event probabilities (the percentage of patients to whom an event applies, e.g. the percentage of patients with LRTI who are on the ICU) by the size of the patient population. Incremental costs per antibiotic day avoided were calculated by dividing the incremental costs per patient by the absolute incremental number of antibiotic days. See S1 and S2 Tables for the calculations.

### Sensitivity & scenario analysis

To test the robustness of the results and to identify the key cost drivers of the model, all parameters were varied by +/- 25%. Additionally, best- and worst-case scenario analyses were conducted by using the lowest and highest differences between usual care and PCT-guided treatment found in the literature for the length of stay and the number of antibiotic days, and, for LRTI, the percentage of patients receiving antibiotics. The lowest and highest values identified in the literature for these parameters were used. In the worst-case scenario, no difference in length of stay (LOS) between PCT-guided ABS and standard care is modelled [36]. For sepsis, a difference in antibiotic days of -3.3 is used [14] and for LRTI of -2.25 [13]. Further, for LRTI patients, no difference in the percentage of patients receiving antibiotics is modelled in the worst-case scenario [16]. For the best-case scenario, the difference in the length of stay on the regular ward is increased to -1.5 days [29], while the ICU stay remains the same as in the base case (i.e. as in the main analysis as described in the methods), since a higher difference was not identified in the literature. The incremental antibiotic days modelled are -6.0 for sepsis and LRTI [15], and the difference in percentage of patients on antibiotics is set at -17%, based on the difference found by Stolz et al. (2009) between 7 and 14 days of antibiotic use [16].

## Results

### Systematic review

**Fig 3:**
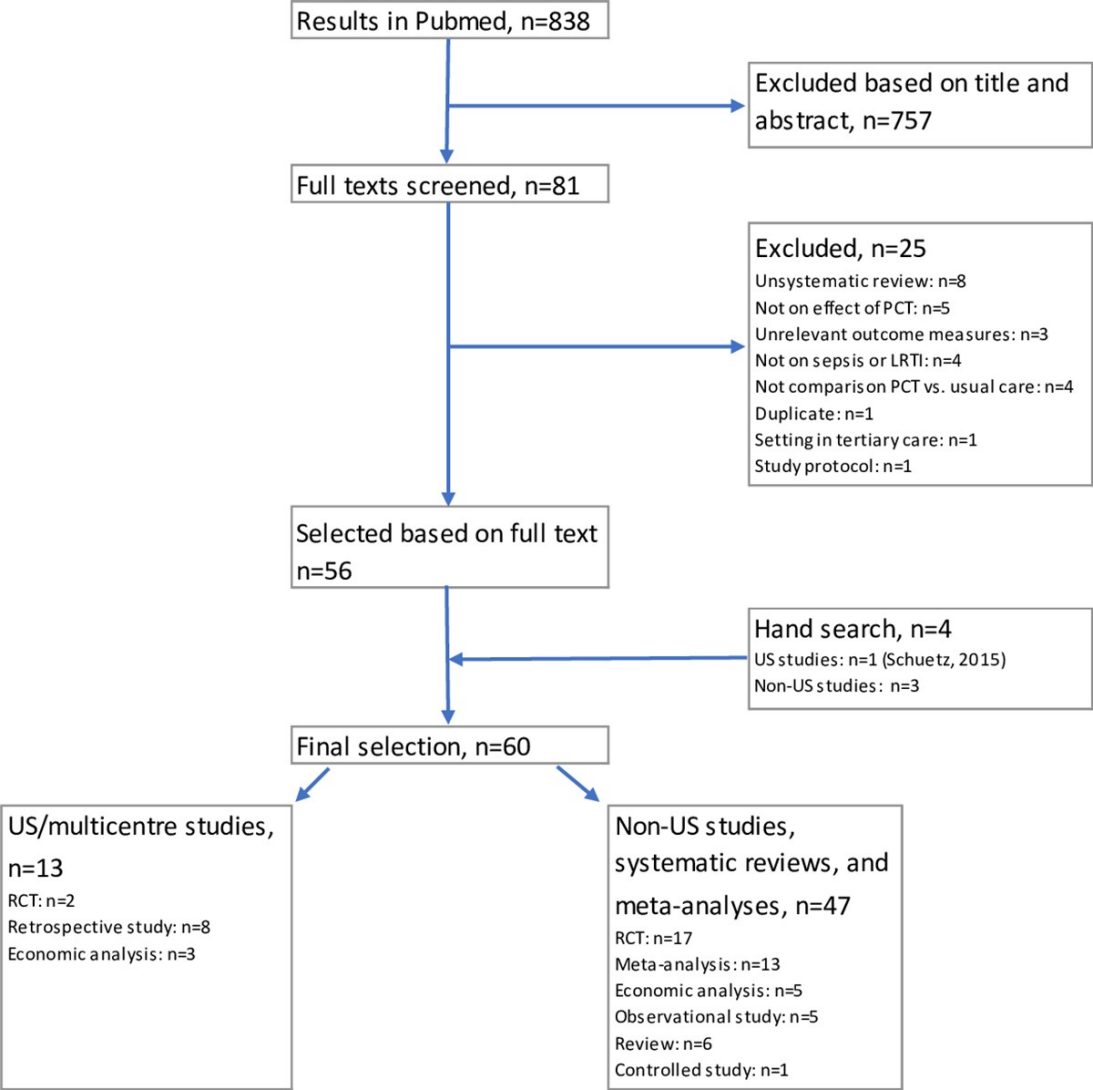
Flow chart of systematic literature review.

The literature search in Pubmed yielded 838 articles. Based on review of titles and abstracts, the full texts of 81 articles were obtained. Of these, 25 articles were excluded. The hand search yielded 4 additional studies resulting in a total selection of 60 articles. Of these, 13 articles reported the results of US-studies [4–7, 13–16, 29, 36–39]. Three of these were multi-country studies including the US [4, 16, 39], and three articles were primarily economic analyses [10–12]. See the flow chart in Fig 3.

### Results for sepsis

Based on the model´s outcomes, PCT-guided ABS reduced the antibiotic duration by 5.83 days in the PCT-group. LOS on the general ward was -0.7 days and on the ICU -3.6 in PCT-guided care versus standard care. The incremental costs (the difference in costs between the two groups) per patient were -$11,311 in the PCT-guided group including productivity losses (Table 4). Therefore, the incremental costs per antibiotic day avoided were -$1,939 (which is the incremental cost per patient divided by the absolute difference in antibiotic days).

**Table 4:** Results for patients with sepsis and LRTI.

	**Outcome**	**Standard care**	**PCT-guided ABS**	**Difference**
**SEPSIS**	***Effectiveness measures***			
	Antibiotic days	13.37	7.54	-5.83
	Patients with antibiotic resistant infection	206,441.76	193,218.28	-13,222.48
	*C*.*difficile* infections	29,374.58	13,271.51	-16,103.07
	***Cost outcomes***			
	Hospital stay	$30,087.16	$22,382.42	-$7,704.75
	Antibiotics	$763.60	$430.52	-$333.18
	Mechanical ventilation	$5,775.00	$3,675.00	-$2,100.00
	Lab tests (including PCT tests in the PCT-group)	$1,711.27	$1,625.63	-$85.64
	Additional costs of antibiotic resistant infection (see S1 Table)	Per patient with ABR: $6,955.31 Per sepsis patient: $1,511.31	Per patient with ABR: $6,786.38 Per sepsis patient: $1,380.15	Per patient with ABR: -$168.94 Per sepsis patient: -$131.16
	Additional costs of *C*.*difficile* infection (see S1 Table)	Per patient with CDI: $11,287.72 Per sepsis patient: $348.99	Per patient with CDI: $11,287.72 Per sepsis patient: $157.68	Per patient with CDI: $0 Per sepsis patient: -$191.32
	Productivity losses per patient	$3,232.90	$2,468.37	-$764.54
	Average total costs per sepsis patient	$43,430.34	$32,119.76	-$11,310.57
	Total costs per sepsis patient population	$41,262,479,518	$30,516,482,033	-$10,745,997,485
	**Outcome**	**Standard care**	**PCT-guided ABS**	**Difference**
**LRTI**	***Effectiveness measures***			
	Antibiotic days	11.90	6.99	-4.91
	Patients with antibiotic resistant infection	369,639.33	305,173.70	-64,465.64
	*C*.*difficile* infections	51,485.59	19,998.90	-31,486.69
	***Cost outcomes***			
	Hospital stay	$9,754.73	$8,149.72	-$1,605.02
	Antibiotics	$585.87	$295.90	-$289.97
	Mechanical ventilation^1^	$606.38	$385.88	-$220.50
	Lab tests (including PCT tests in the PCT-group)	$1,292.32	$1,361.80	$69.48
	Additional costs of antibiotic resistant infection (see S2 Table)	Per patient with ABR: $11,139.02 Per LRTI patient: $2,168.45	Per patient with ABR: $11,125.20 Per LRTI patient: $1,788.05	Per patient with ABR: -$13.82 Per LRTI patient: -$380.40
	Additional costs of *C*.*difficile* (see S2 Table)	Per patient with CDI: $11,287.72 Per LRTI patient: $306.07	Per patient with CDI: $11,287.72 Per LRTI patient: $118.89	Per patient with CDI: $0 Per LRTI patient: -$187.18
	Productivity losses per patient	$1,503.84	$1,250.50	-$253.34
	Average total costs per LRTI patient	$16,217,65	$13,350.73	-$2,866.92
	Total costs per LRTI patient population	$30,793,879,222	$25350,197,961	-$5,443,681,261

All results are rounded to two digits. Numbers not adding up are due to rounding.

ABR = Antibiotic resistance, CDI = *C*.*difficile* infection

On the patient population level, the incremental costs for PCT-guided care were -$10,745,997,485. The number of sepsis patients with antibiotic resistant infections in hospital was estimated to be 6.4% lower in PCT-guided care than in standard care, with 206,442 versus 193,219 patients, respectively. The number of hospital *C*.*difficile* infections in PCT-guided care was estimated to be 54.8% lower than in standard care, with 13,272 versus 29,375 *C*.*difficile* infections, respectively. See Table 4.

### Results for LRTI

In the PCT-group, the number of antibiotic days avoided was 4.91 per patient. LOS on the general ward was -0.7 days and on the ICU -3.6 in PCT-guided care versus standard care. The incremental total costs (the difference in costs between the two groups) per LRTI patient, including productivity losses, were -$2,867. Therefore, the incremental costs per antibiotic day avoided were -$584 (which is the incremental cost per patient divided by the absolute difference in antibiotic days).

For the whole LRTI patient population the incremental total costs were -$5,443,681,261. The number of patients with ABR in hospital was estimated to be 17.4% lower in PCT-guided care with 305,174 resistant infections compared to 369,639 resistant infections in standard care. The number of patients with hospital *C*.*difficile* infection in standard care was estimated to be 51,486 and 19,999 in PCT-guided care, which is a reduction of 61.2%. See Table 4.

### Sensitivity & scenario analysis

**Fig 4:**
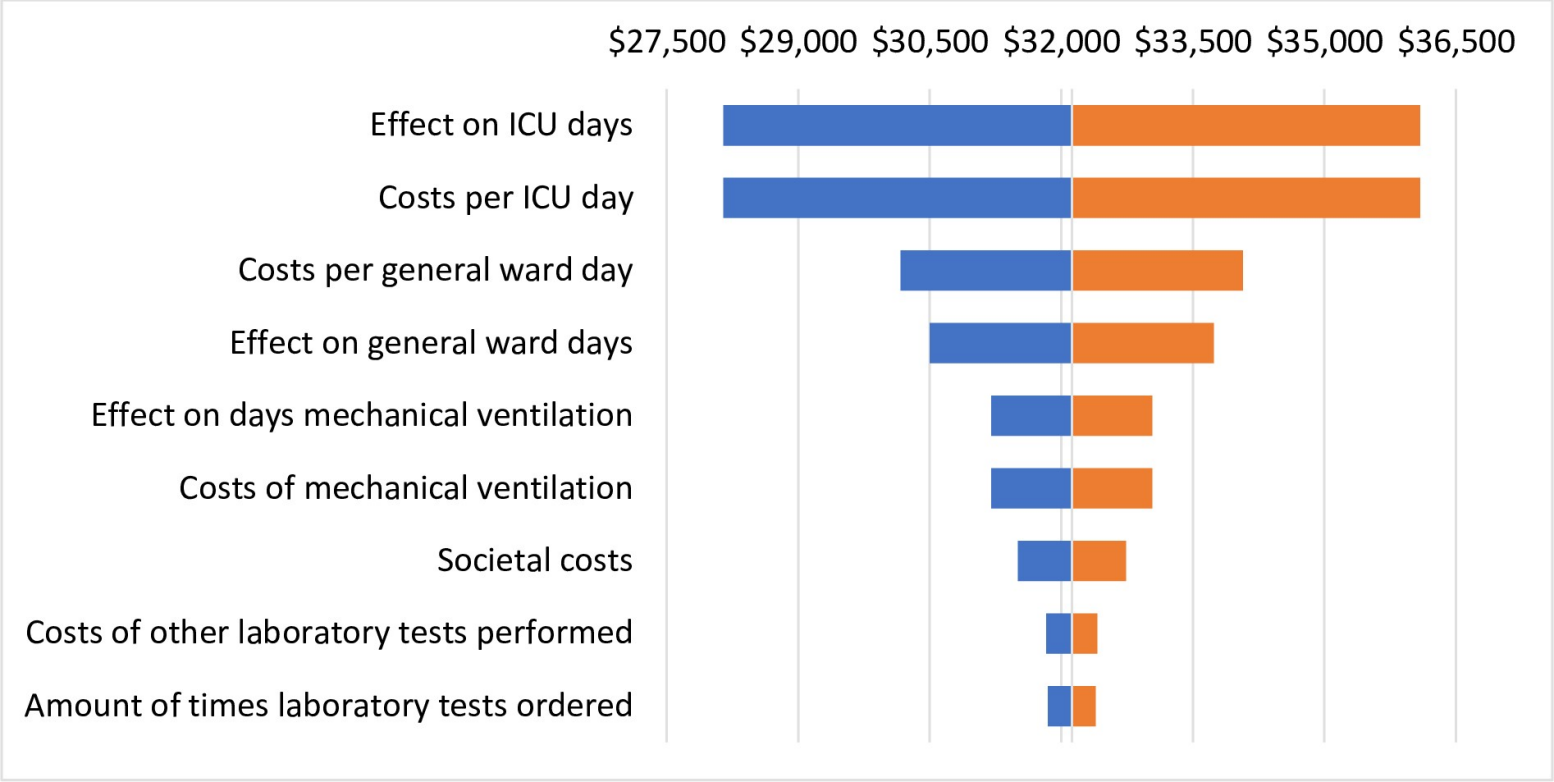
Tornado diagram showing the results of the sensitivity analysis for sepsis on the total costs per patient in comparison to the base case values of the main analysis. The tornado diagram shows to what degree changes in an input variable influence the total costs of PCT-guided care. While all other parameters are being held equal, the value of one input parameter is varied by + or– 25%. The tornado diagram shows the difference of this result to the result of the primary analysis, in which the values as defined in the input parameter tables are used.

**Fig 5:**
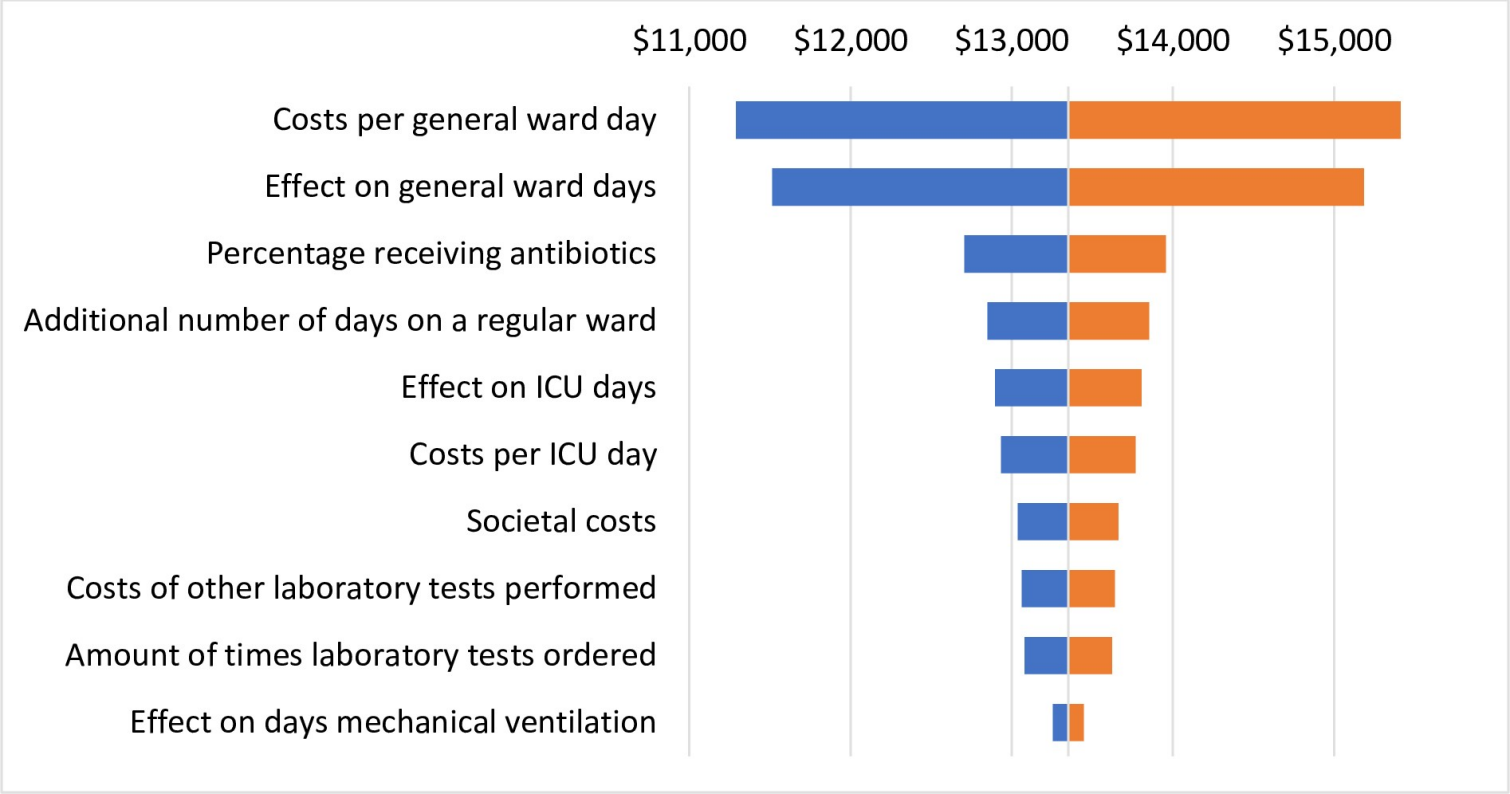
Tornado diagram showing the results of the sensitivity analysis for LRTI on the total costs per patient in comparison to the base case values of the main analysis.

The one-way sensitivity analysis showed that the model results for the sepsis population are most sensitive to 1) effect on ICU days and costs per ICU day, 2) the costs per general ward day, and 3) the effect on general ward days. For LRTI, these were 1) the cost per general ward day, 2) the effect on general ward days, and 3) the percentage of patients receiving antibiotics. The tornado diagrams in Figs 4 and 5 show the ten most influential parameters for sepsis and LRTI, respectively.

In the worst-case scenario, for both sepsis and LRTI, the total incremental costs remained in the cost-saving range, with -$3,416 and -$554, respectively. These cost savings resulted from fewer days on antibiotics, leading to lower costs for antibiotics and fewer patients with ABR and *C*.*difficile* infections. In the best-case scenario, the cost savings increased to -$12,345 for sepsis and to -$4,201 or LRTI.

## Discussion

We evaluated the comparative effectiveness and the costs of PCT-guided ABS versus usual care for sepsis and LRTI patients from the US societal perspective. To the best of our knowledge this is the first cost analysis that is mainly based on US studies. For both patient groups, PCT-guided ABS is expected to reduce antibiotic exposure and to lead to substantially lower societal costs. In addition, the model projected reductions in the number of patients with ABR and *C*.*difficile* infections.

The incremental total costs, including treatment costs and productivity losses, amounted to -$11,311 per sepsis patient, implying cost-savings in the PCT-group. For LRTI patients, the incremental total costs were -$2,867 per patient in the PCT-group compared to usual care. Given the large numbers of sepsis and LRTI patients, the yearly cost-savings from the US perspective are substantial; -$10.7billion for sepsis and -$5.4billion for LRTI. Projected reductions in ABR and *C*.*difficile* infections were 6.4% and 54.8% for sepsis and 17.4% and 61.2% for LRTI, respectively. The cost-savings were mainly driven by the reduction in LOS and the reduced number of patients with ABR in patients with LRTI. For patients with sepsis, savings were driven by a shorter LOS and reduced costs for mechanical ventilation. The results of the scenario analysis showed that even in the most conservative scenarios, cost-savings were realised.

Previous economic and cost analyses have been conducted for the US of PCT to guide antibiotic therapy. The study by Harrison et al. (2015) [7] analysed the cost impact of PCT for adult ICU patients suspected of sepsis or bacterial infection from the US hospital perspective, with effectiveness data (among others) being based on a French study, reviews, and meta-analyses. The total costs in standard care were $40,663 versus $40,597 in PCT-guided ABS, with cost-savings of $65 in PCT. This study found comparable total costs to our results, but a much smaller cost difference. The larger cost difference in our study is caused by the difference in LOS that we included in our model.

Smith et al. (2013) [6] evaluated the costs per quality-adjusted life year for patients hospitalised with community-acquired pneumonia. Different from our analysis, only antibiotic use and antibiotic costs were assumed to differ between the PCT and the standard care group. It was found that the costs in the PCT group were $22 higher than in usual care. Sensitivity analyses showed that one of the parameters with most influence on total costs is decreased hospital length of stay, which was similar to our results.

The economic evaluation by Schuetz et al. (2015) [2] assessed the cost-effectiveness of PCT-guided ABS for patients with acute respiratory tract infections from the perspective of a US integrated delivery network. The effectiveness data was based on a meta-analysis and adapted to fit the US-situation. For PCT-guided care, substantial cost-savings of $700,000 were found for a 1m-member catchment-area, supporting our result of cost savings in the PCT-guided group.

For the US, a limited amount of research on the effectiveness of PCT-guided ABS was available. The FDA, for example, cleared high-sensitive PCT for the management of antibiotics in LRTI and sepsis based on a meta-analysis of several European trials, which showed a safe reduction in antibiotic days of -3.2 and a change in length of stay of 1.1 days on the ICU (an increase in the PCT-group) and -1.4 days on the general ward. Still, in our analysis, all but one of the most influential input parameters (according to the sensitivity analysis) were based on US data.

Huang et al. recently published the ProACT study and found no difference for the number of antibiotic days and the number of adverse events [40]. It should be noted that the patient population of the ProACT study differs from the patients with LRTI in our analysis. Whereas we included only hospitalised patients with LRTI, less than half (47%) of the ProACT patients were hospitalised. Further, a third of included patients had a diagnosis of asthma, a non-LRTI where antibiotics are not generally indicated.

Our paper has limitations regarding the data that was used in the model. The data underlying the main parameters in our model were based on either one or a few studies. As a systematic literature review was conducted to identify all data available and suitable for the model, the results show the cost-savings that can be expected given the best evidence available at that time. The study by Bishop et al. (2014) [14], e.g., included 100 participants. Some larger US-studies that were identified in our literature review unfortunately did not provide data that are required for a health economic analysis. For example, while our analysis compared PCT-guided therapy to standard care, many studies compared PCT algorithm-adherent patients to algorithm non-adherent patients [5] or patients with a PCT-value of <0.25ng/mL to patients with a PCT-value ≥0.25ng/mL [36–39]. Regarding antibiotic use, we included the number of antibiotic days in our model, whereas many studies provided the percentage of patients that received antibiotics for longer than 48 hours [36], presented the results as an adjusted risk ratio [37], or summarised results for all patients [38]. In addition, the retrospective study by Balk et al. (2017) [29] was not included in the effectiveness analysis because patients with serial PCT testing after the first day of ICU care were not included in this database, and therefore “it is unlikely that PCT was used to guide early antibiotic discontinuation” (p.32), which was the focus of our model notably for the sepsis population.

For some parameters, data was not available specifically for the US and therefor based on European data. All non-US data used in the model were validated for their applicability to the US healthcare setting by the four clinical authors of this study. From a multi-country study, the US-specific data was requested but not received. The change in antibiotic resistance infections, depending on the change in antibiotic days, was based on two studies. As long-term data are lacking, we conservatively projected the impact on ABR solely based on the reduction in days of AB exposure and LOS. Mortality was not included in our model; however, it should be noted that multiple RCTs established PCT being safe [29]. In a recent, large patient-level meta-analyses including more than 6,700 patients a significant reduction in 30-day mortality was found for PCT use compared to controls [4]. Finally, our model does neither consider the impact of shorter antibiotic duration on bloodstream infections through IV-administration nor include quality-adjusted life years.

Further, while the study by Bishop et al. (2014) [14] is one of the few US studies to date, it presents results for patients with sepsis and patients with LRTI combined.

For analysing the cost impact of PCT using data that is completely based on patients with the specific disease, future research should present the results of patients with different diseases separately. Still, the model is based on the best evidence that is currently available. In addition, more US-based research on the effect of PCT-testing is needed to include the results of more studies in the model. Finally, the long term impact of PCT on ABR and *C*.*difficile* infections, and the impact on other outcomes such as bloodstream infections and health-related quality of life need to be studied.

## Conclusion

Our results showed that using a PCT-algorithm to guide antibiotic use in sepsis and hospitalised patients with lower respiratory tract infections reduced the total costs, including treatment costs and productivity losses, in the US by $11,311 and $2,867, respectively. The cost drivers were shown to be the patients´ length of stay, the costs of the hospital stay, and, for patients with LRTI, the percentage of patients receiving antibiotics. The number of patients with antibiotic resistance infections and *C*.*difficile* infections were reduced considerably, however, this was based on very few studies. Considering the growing public health concern related to antibiotic resistance infections and *C*.*difficile* infections, future investigations should focus on impact of PCT-guided ABS on the number of patients with antibiotic resistance infection and *C*.*difficile* infection.

## Supporting information

S1 Table Cost calculation for sepsis. (DOCX)

S2 Table Cost calculation for LRTI. (DOCX)

## Abbreviations

PCT: Procalcitonin
LRTI: Lower respiratory tract infection
ABS: Antibiotic stewardship
ABR: Antibiotic resistance
ICU: Intensive care unit
CAP: Community-acquired pneumonia
VAP: Ventilator-associated pneumonia
LOS: Length of stay
ABX: Antibiotics
