# Supplementary material for: The cost impact of PCT-guided antibiotic stewardship versus usual care for hospitalised patients with suspected sepsis or lower respiratory tract infections in the US: A health economic model analysis
Source: PLoS One. 2019 Apr 23;14(4):e0214222. doi: 10.1371/journal.pone.0214222 (PMC6478294; doi:10.1371/journal.pone.0214222)
Supplement: S1 Table — (DOCX) [file pone.0214222.s001.docx]

Supplemental Table 1: Cost calculation for sepsis

|  | **Standard care** | **PCT-guided care** | **Incremental costs** |
| --- | --- | --- | --- |
| **Hospital stay costs** | Costs general ward + Costs ICU:  5.8 days on general ward*$1,270.58+12 days on ICU*$1,893.15=$30,087.16 | Costs general ward + Costs ICU:  5.1 days on general ward*$1,270.58+8.4 days on ICU*$1,893.0.15=$22,382.41 | $-7,704.75 |
| **Costs of antibiotics** | 13.37^a^ days on antibiotics*$57.13=$763.70 | 7.54^a^ days on antibiotics*$57.13=$430.52 | -$333.18 |
| **Costs of mechanical ventilation** | 5.5 days on mechanical ventilation*$1,050.00 =$5,775.00 | 3.5 days on mechanical ventilation*$1,050.00 =3,675.00 | -$2,100.00 |
| **Costs of blood cultures** | 2 blood cultures taken*0.975 of patients having their blood culture taken*$19.14*(1/0.0818 of patients with blood culture performed diagnosed as having sepsis) =$456.27 | 2 blood cultures taken*0.614 of patients having their blood culture taken*$19.14*(1/0.0818 of patients with blood culture performed diagnosed as having sepsis) =$287.33 | -$168.94 |
| **Costs of PCT tests** | 0 PCT tests*$49.66=0 | 5 PCT tests*$49.66=$248.30 | $248.30 |
| **Costs of laboratory tests** | 25.1 lab tests*$50.00=$1,255.00 | 21.8 lab tests*$50=$1,090.00 | -$165.00 |
| **Additional costs of antibiotic resistance infection per patient with sepsis** | - Additional costs for a prolonged stay (4.6 days on the general ward with isolation of $50 extra) per patient with ABR infection =$6,074.67 - Additional costs of blood cultures and lab tests taken per patient with ABR infection= $880.65     Additional total costs per patient with ABR infection=$6,074.67+$880.60=$6,955.31    Additional costs of antibiotic resistance for all patients=$6,955.31*950,074 patients with sepsis in the US per year*21.7%^a^ of patients developing antibiotic resistance infection  =$1,435,876,336    Additional cost of antibiotic resistance per patient with sepsis=$1,435,876,336^a^/950,074 patients with sepsis in the US per year =$1,511.31 | - Additional costs for a prolonged stay (4.6 days on the general ward with isolation of $50 extra) per patient with ABR infection=$6,074.67 - Additional costs of blood cultures and lab tests taken per patient with ABR infection= $711.71     Additional total costs per patient with ABR infection=$6,074.67+$711.71=$6,786.38    Additional costs of antibiotic resistance for all patients=$6,786.38*950,074 patients with sepsis in the US per year*(21.7%^a^ of patients developing antibiotic resistance infection -(3.2%^a^ reduction in antibiotic resistance infection*43.6%^a^ reduction in antibiotic days))  =$1,311,258,863    Additional cost of antibiotic resistance per patient with sepsis= $1,311,258,863^a^/950,074 patients with sepsis in the US per year = $1,380.15 | -$131.16 |
| **Additional costs of *C.difficile* infection per patient with sepsis** | - Additional costs for a prolonged stay (8.49 days on the general ward with isolation of $50 extra) per patient with *C.difficile* infection=$11,208.42 - Additional costs for diagnostic tests taken per patient with *C.difficile* infection = $79.30     Additional total costs per patient with *C.difficile* infection =$11,208.42+$79.30=$11,287.72    Additional costs of *C.difficile* infection for all patients=$11,287.72*3.1%^a^ developing CDI*950,074 patients with sepsis in the US per year  =$331,572,194.55    Additional cost of *C.difficile* infection per patient with sepsis=$331,572,194.55/950,074 patients with sepsis in the US per year =$348.99 | - Additional costs for a prolonged stay (8.49 days on the general ward with isolation of $50 extra) per patient with *C.difficile* infection=$11,208.42 - Additional costs of diagnostic tests taken per patient with *C.difficile* infection = $79.30     Additional total costs per patient with *C.difficile* infection =$11,208.42+$79.30=$11,287.72    Additional costs of *C.difficile* infection for all patients=$11,287.72*1.4%^a^ developing CDI*950,074 patients with sepsis in the US per year  =$149,805,175.72    Additional cost of *C.difficile* infection per patient with sepsis=$149,805,175.72/950,074 patients with sepsis in the US per year =$157.68 | -$191.32 |
| **Productivity losses** | Costs of missed days of work (17.8 days of 8h valued at $21.20)+ costs of missed days of work due to antibiotic resistance per patient with sepsis (4.6 additional days of 8h valued at $21.20 applied to patients with antibiotic resistance infection and averaged out over the whole patient population) + costs of missed days of work due to *C.difficile* infection per patient with sepsis (8.49 additional days of 8h valued at $21.20 applied to patients with CDI and averaged out over the whole patient population)  $3,018.88+$169.52^a^+$44.51^a^=$3,232.90^a^ | Costs of missed days of work (13.5 days of 8h valued at $21.20) + costs of missed days of work due to antibiotic resistance per patient with sepsis (4.6 additional days of 8h valued at $21.20 applied to patients with antibiotic resistance infection and averaged out over the whole patient population) + costs of missed days of work due to *C.difficile* infection per patient with sepsis (8.49 additional days of 8h valued at $21.20 applied to patients with CDI and averaged out over the whole patient population)  $2,289.60+$158.66^a^+$20.11^a^=$2,468.37^a^ | -$764.54^a^ |
| **Total costs per patient** | $30,087.16 for the hospital stay  +$763.70 for antibiotics^a^  +$5,775.00 for mechanical ventilation  +$456.27 for blood cultures^a^  +0$ for PCT tests  +$1,255.00 for other laboratory tests  +$1,511.31 for antibiotic resistance / sepsis patient^a^  +$348.99 for *C.difficile* infection / sepsis patient^a^  +$3,232.90 for productivity losses^a^  =$43,430.34^a^ | $22,382.41 hospital stay costs  +$430.52 for antibiotics^a^  +3,675.00 for mechanical ventilation  +$287.33 for blood cultures^a^  +$248.30 for PCT tests  +$1,090.00 for other laboratory tests  +$1,380.50 for antibiotic resistance / sepsis patient^a^  +$157.68 for *C.difficile* infection / sepsis patient^a^  +$2,468.37 for productivity losses^a^  =$32,119.76^a^ | **-$11,310.57** |
| **Total costs for the yearly sepsis population** | Total costs per patient of $43,430.34^a^*950,074 patients with sepsis in the US per year  =$41,262,479,518 | Total costs per patient of $32,119.76^a^*950,074 patients with sepsis in the US per year  =$30,516,482,033 | **-$10,745,997,485** |

CDI=*C.difficile* infection

^a^) rounded
