# Supplementary material for: The cost impact of PCT-guided antibiotic stewardship versus usual care for hospitalised patients with suspected sepsis or lower respiratory tract infections in the US: A health economic model analysis
Source: PLoS One. 2019 Apr 23;14(4):e0214222. doi: 10.1371/journal.pone.0214222 (PMC6478294; doi:10.1371/journal.pone.0214222)
Supplement: S2 Table — (DOCX) [file pone.0214222.s002.docx]

Supplemental Table 2: Cost calculation for LRTI

|  | **Standard care** | **PCT-guided care** | **Incremental costs** |
| --- | --- | --- | --- |
| **Hospital stay costs** | Costs patients without ICU stay + costs patients with ICU stay:  5.8 days on the general ward*$1,270.58 + 12.0 days on the ICU*10.5% of patients being admitted to the ICU*$1,893.15 =$9,754.73 | Costs patients without ICU stay + costs patients with ICU stay:  5.1 days on the general ward*$1,270.58 + 8.4 days on the ICU*10.5% of patients being admitted to the ICU*$1,893.15 =$8,149.72 | $-1,605.02 |
| **Costs of antibiotics** | 11.90^a^ days on antibiotics*$56.16*87.7% on antibiotics=$585.87 | 6.99^a^ days on antibiotics*$56.16*75.4% on antibiotics=$295.90 | -$289.97^a^ |
| **Costs of mechanical ventilation** | 5.5 days on mechanical ventilation*10.5% of patients being admitted to the ICU*$1,050.50 =$606.38 | 3.5 days on mechanical ventilation*10.5% of patients being admitted to the ICU*$1,050.50 =$385.88 | -$220.50 |
| **Costs of blood cultures** | 2 sets of blood cultures taken*97.5% of patients having blood cultures taken*$19.14=$37.32 | 2 sets of blood cultures taken*61.4% of patients having blood cultures taken*$19.14=$23.50 | -$13.82 |
| **Costs of PCT tests** | 0 PCT tests*$49.66=0 | 5 PCT tests*$49.66=$248.30 | $248.30 |
| **Costs of laboratory tests** | 25.1 lab tests*$50.00=$1,255.00 | 21.8 lab tests*$50=$1,090.00 | -$165.00 |
| **Additional costs of antibiotic resistance infection per patient with LRTI** | - Additional costs for a prolonged stay (8.1 days on the general ward including $50 for a stay in isolation) per patient with ABR infection=$10,696.70 - Additional costs of blood cultures and lab tests taken per patient with ABR infection= $442.32   Additional total costs per patient with ABR infection=$10,696.70+442.32=$11,139.02    Additional costs of antibiotic resistance infection for all patients=  $11,139.02*1,898,788 patients with LRTI in the US per year*87.7% of patients taking antibiotics*22.2%^a^ of patients developing antibiotic resistance infection  =$4,117,420,312^a^    Additional cost of antibiotic resistance infection per patient with LRTI=$4,117,420,312^a^/1,898,788 patients with LRTI in the US per year =$2,168.45^a^ | - Additional costs for a prolonged stay (8.1 days on the general ward including $50 for a stay in isolation) per patient with ABR infection=$10,696.70 - Additional costs of blood cultures and lab test taken per patient with ABR infection= $428.50   Additional total costs per patient with ABR infection=$10,696.70+328.50=$11,125.20    Additional costs of antibiotic resistance infection for all patients=  $11,125.20*1,898,788 patients with LRTI in the US per year* 75.4% of patients taking antibiotics *(22.2%^a^ of patients developing antibiotic resistance infection - ((3.2%^a^ reduction in antibiotic resistance infection *0.413^a^ reduction in antibiotic duration) +(3.2%^a^*0.140 reduction in antibiotic prescriptions)) /2))=$3,395,118,998^a^    Additional cost of antibiotic resistance infection per patient with LRTI= $3,395,118,998^a^/1,898,788 patients with LRTI in the US per year = $1,788.05^a^ | -$380.40^a^ |
| **Additional costs of *C.difficile* infection per patient with LRTI** | - Additional costs for a prolonged stay (8.49 days on the general ward including $50 for a stay in isolation) per patient with *C.difficile* infection=$11,208.42 - Additional costs for diagnostic tests taken per patient with *C.difficile* infection = $79.30     Additional total costs per patient with *C.difficile* infection =$11,208.42+$79.30=$11,287.72    Additional costs of *C.difficile* infection for all patients=$11,287.72*3.1%^a^ developing CDI*87.7% of patients taking antibiotics*1,898,788 patients with LRTI in the US per year=$581,155,074^a^    Additional cost of *C.difficile* infection per patient with LRTI=$581,155,074^a^/1,898,788 patients with LRTI in the US per year=$306.07^a^ | - Additional costs for a prolonged stay (8.49 days on the general ward including $50 for a stay in isolation) per patient with *C.difficile* infection=$11,208.42 - Additional costs of blood cultures taken per patient with *C.difficile* infection = $79.30     Additional total costs per patient with *C.difficile* infection =$11,208.42+$79.30=$11,287.72    Additional costs of *C.difficile* infection for all patients=$11,287.72*1.4%^a^ developing CDI*75.4% of patients taking antibiotics*1,898,788 patients with LRTI in the US per year=$225,742,069^a^    Additional cost of *C.difficile* infection per patient with LRTI=$225,742,069^a^/1,898,788 patients with LRTI in the US per year=$118.89^a^ | -$187.18^a^ |
| **Productivity losses** | Costs of missed days of work (days on the general ward and ICU (if applicable) of 8h valued at $21.20 an hour) + costs of missed days of work due to antibiotic resistance per patient with LRTI (8.1 additional days of 8h valued at $21.20 applied to patients with antibiotic resistance infection and averaged out over the whole patient population) + costs of missed days of work due to *C.difficile* infection per patient with LRTI (8.49 additional days of 8h valued at $21.20 applied to patients with CDI and averaged out over the whole patient population)  $1,197.38+$276.43^a^+$39.03^a^=$1,504.84^a^ | Costs of missed days of work (days on the general ward and ICU (if applicable) of 8h valued at $21.20 an hour) + costs of missed days of work due to antibiotic resistance per patient with LRTI (8.1 additional days of 8h valued at $21.20 applied to patients with antibiotic resistance infection and averaged out over the whole patient population) + costs of missed days of work due to *C.difficile* infection per patient with LRTI (8.49 additional days of 8h valued at $21.20 applied to patients with CDI and averaged out over the whole patient population)  $1,015.55+$220.79^a^+$15.16^a^=$1,250,50^a^ | -$253.34^a^ |
| **Total costs per patient** | $9,754.73 for the hospital stay  +$585.87 for antibiotics^a^  +$606.38 for mechanical ventilation  +$1,292.32 for lab costs  +$2,168.45 for antibiotic resistance infections^a^  +$306.07 for CDI infections^a^  +$1,503.84 for productivity losses^a^  =$16,217.65^a^ total costs per patient | $8,149.72 for the hospital stay  +$295.90 for antibiotics^a^  +$385.88 for mechanical ventilation  +$1,361.80 for lab costs  +$1,788.05 for antibiotic resistance infections^a^  +$118.89 for CDI infections^a^  +$1,250.50 for productivity losses^a^  =$13,350.73^a^ total costs per patient | **-$2,866.92^a^** |
| **Total average costs for the yearly LRTI population** | $16,217.65^a^ total average costs per patient with sepsis*1,898,788 patients with LRTI in the US per year=$30,793,879,222^a^ | $13,350.73^a^ total average costs per patient with sepsis*1,898,788 patients with LRTI in the US per year=$25,350,197,961^a^ | **-$5,443,681,261^a^** |

CDI=*C.difficile* infection

^a^) rounded
